# Position Validation of the Dwarfing Gene *Dw6* in Oat (*Avena sativa* L.) and Its Correlated Effects on Agronomic Traits

**DOI:** 10.3389/fpls.2021.668847

**Published:** 2021-05-20

**Authors:** Honghai Yan, Kaiquan Yu, Yinghong Xu, Pingping Zhou, Jun Zhao, Ying Li, Xiaomeng Liu, Changzhong Ren, Yuanying Peng

**Affiliations:** ^1^Triticeae Research Institute, Sichuan Agricultural University, Chengdu, China; ^2^State Key Laboratory of Crop Gene Exploration and Utilization in Southwest China, Sichuan Agricultural University, Chengdu, China; ^3^Baicheng Academy of Agricultural Sciences, Baicheng, China

**Keywords:** oat, dwarfing gene, *Dw6*, genetic effect, mapping

## Abstract

An F_6__:__8_ recombinant inbred line (RIL) population derived from the cross between WAOAT2132 (*Dw6*) and Caracas along with the two parents were used to evaluate the genetic effects of *Dw6* dwarfing gene on plant height and other agronomic traits in oat (*Avena sativa* L.) across three environments, and develop closely linked markers for marker-assisted selection (MAS) for *Dw6*. The two parents differed in all investigated agronomic traits except for the number of whorls. The RIL lines showed a bimodal distribution for plant height in all three tested environments, supporting the height of this population was controlled by a single gene. *Dw6* significantly reduced plant height (37.66∼44.29%) and panicle length (13.99∼22.10%) but without compromising the coleoptile length which was often positively associated with the reduced stature caused by dwarfing genes. *Dw6* has also strong negative effects on hundred kernel weight (14.00∼29.55%), and kernel length (4.21∼9.47%), whereas the effects of *Dw6* on the kernel width were not uniform across three environments. By contrast, lines with *Dw6* produced more productive tillers (10.11∼10.53%) than lines without *Dw6*. All these together suggested the potential yield penalty associated with *Dw6* might be partially due to the decrease of kernel weight which is attributed largely to the reduction of kernel length. Eighty-one simple sequence repeat (SSR) primer pairs from chromosome 6D were tested, five of them were polymorphic in two parents and in two contrasting bulks, confirming the 6D location of *Dw6*. By using the five polymorphic markers, *Dw6* was mapped to an interval of 1.0 cM flanked by markers SSR83 and SSR120. Caution should be applied in using this information since maker order conflicts were observed. The close linkages of these two markers to *Dw6* were further validated in a range of oat lines. The newly developed markers will provide a solid basis for future efforts both in the identification of *Dw6* in oat germplasm and in the determination of the nature of the gene through positional cloning.

## Introduction

The cultivated oat (*Avena sativa* L.) is the sixth important cereal crop that is widely used as both animal feed and human food. Its cropped area, however, has rapidly declined during the past decades (FAOSTATS, 2019), even the demand in oat for human consumption has increased in recent years due to its documented health benefits. This is partly attributed to the lower yield of oat compared with that of the other cereal crops (FAOSTATS, 2019; Yan et al., 2020). Thus, breeding high-yielding oat varieties are urgently needed to turn oats more competitive and attractive for farmers.

Plant height is an important agronomic trait that is highly related to lodging, a major problem of oat (Zimmer et al., 2018). In wheat, the introduction of the dwarfing genes into commercial varieties has reduced the lodging risk and greatly increased the yield of wheat, and led to widespread adoption of the dwarfing genes throughout the world (Gale and Youssefian, 1985). Similar strategies have been used in oat breeding programs. Until present, eight dwarfing genes have been officially reported in oat (Zhao et al., 2018), however, only a few including *Dw6*, *Dw7*, and *Dw8* are present in readily available germplasm (Molnar et al., 2012). Of these, *Dw6* is the most extensively used gene that has been introduced into commercial oat varieties (Milach and Federizzi, 2001).

*Dw6* was derived from oat line OT184 through fast-neutron irradiation (Brown et al., 1980). It is a dominant and gibberellin (GA) sensitive gene that conditioning semi-dwarfness (Brown et al., 1980; Milach et al., 2002). Mutation line with *Dw6* allele has a normal internode number but with a significant reduction in length of the highest three internodes compared with its non-dwarf counterpart (Milach et al., 2002). The *Dw6* allele could reduce up to 37% of the plant height on average in some cultivation environments (Milach et al., 2002). Though the effects of *Dw6* on oat plant height have been extensively investigated, there has been only a small amount of work exploring the potential yield improvement offered by *Dw6*, or its effects on other important traits. Field experiments in Australia showed oat cultivars with *Dw6* have outyielded taller types in most regions of Australia (Barr, 1984; Anderson and McLean, 1989), and have rapidly replaced the formerly widely grown tall cultivars (Anderson and McLean, 1989). However, such an increase in yield has not been observed in other studies. Marshall et al. (1987) observed the conventional height cultivars have a higher yield than the dwarf ones at different management levels in Pennsylvania. After examining the effects of *Dw6* on yield and agronomic features that are closely related to yield of oat at three locations in central Alberta, Canada in three consecutive cropping seasons, Kibite and Clayton (2000) reported a significant negative effect of *Dw6* on grain yield, test weight, and kernel weight.

Indeed, many reported dwarf genes in crops have negative effects on yield and (or) yield components. Short coleoptile and low early seedling vigor associated with some dwarf genes, particularly these GA-insensitive genes, such as wheat *Rht-B1b*, *Rht-B1c*, and *Rht-D1b*, are the likely reasons for the lower yields in an adverse environment (e.g., deep sowing) (Allan, 1989; Rebetzke et al., 1999; Addisu et al., 2009). Other studies showed some dwarfing genes negatively affected the grain yield by decreasing the grain size (Chen et al., 2013), kernel thousand weight (Kantarek et al., 2018), or other yield components (Kowalski et al., 2016). However, the yield penalty caused by *Dw6* has been assumed to be the failure of the panicle to fully emerge from the leaf sheath (Milach and Federizzi, 2001).

Despite these challenges, the *Dw6* gene has been considered as the most potential gene resource to breed dwarf varieties with good yield (Milach and Federizzi, 2001; Tanhuanpää et al., 2006). Efforts have been made to map *Dw6* gene by using different mapping populations (Milach et al., 1997; Tanhuanpää et al., 2006; Molnar et al., 2012; Zhao et al., 2018) and finally located it on chromosome 18D by comparative analysis (Zhao et al., 2018). However, to be partially limited by the number of available markers, the associated markers are still genetically far away from *Dw6* (The closest marker is 1.2 cM distant from *Dw6*). Recently, great progress has been made in the development of a high-quality hexaploid oat reference genome by using a complementary approach combining short and long-read DNA sequencing technologies^1^. This will greatly facilitate the development of DNA markers, identification of genes underlying agronomic traits and other genomic research in oat.

The objectives of this study were to evaluate the effects of *Dw6* on plant height and some other yield-related agronomic traits, including panicle length, number of whorls (branches) on the main tiller, spikelet number per panicle, productive tiller number, kernel weight, kernel size (length, width and perimeter) and coleoptile length under subtropical growth conditions, and to develop new SSR marker using the recently published hexaploid oat reference genome for further mapping *Dw6* using a RIL population.

## Materials and Methods

### Plant Materials

A cross was generated between an early, high-quality and *Dw6*-containing line WAOAT2132, with another early, high-quality but tall oat line Caracas. The self-pollinated F_1_ produced 306 F_2_ progenies (Zhao et al., 2018). Further self-pollination without selection for four generations resulted in F_5__:__6_ individuals. Seeds from these lines were threshed and then sown in the autumn of 2016 into rows for plant height assessment. A single panicle was harvested from each of 269 lines homogenous for plant height and increased during the 2017–2018 cropping season to produce an F_2_-derived, F_6__:__8_ recombination inbred lines (RILs). This F_6__:__8_ WAOAT2132/Caracas RIL population was used for mapping the *Dw6* locus in this study (Supplementary Table 1). DNA samples from other materials used in previous work were also used to test markers linked to *Dw6* in this study (Supplementary Table 2). These included 44 diverse tall lines from 13 countries, four semi-dwarf lines [three of them, OT207, Potoroo and AC Ronald, have been reported to have *Dw6* (Mitchell Fetch et al., 2003; Zhao et al., 2018)], and 14 near-isogenic lines (NILs) contrasting for the presence of *Dw6* provided by Molnar et al. (2012).

### Field Trials

The F_6__:__8_ RILs and its parents were grown at Wenjiang (103°51′E, 30°43′N) and Chongzhou (103°38′E, 30°32′N) during 2018–2019 cropping season and at Wenjiang (103°51′E, 30°43′N) during 2019–2020 cropping season. Field trials were arranged in randomized complete blocks with no replication at each location. Each plot comprised two 1 m rows with 20 plants in each row and spaced 30 cm apart. Early one week of sowing, nitrogen and superphosphate fertilizers were applied at a ratio of 80 kg/ha. To avoid water stress, supplemental irrigation was provided as needed. While other field managements were following local standard practices (Yu et al., 2018).

### Assessment of Agronomic Traits

Plant height and other yield-related traits including panicle length, number of whorls (branches) on the main tiller, spikelet number per panicle, and productive tiller number were evaluated in all three environments with the exception of productive tiller number which was not counted in the 2019–2020 cropping season. For measurements of the abovementioned traits, five representative plants in each line from the middle part of the row were chosen, and the mean values of each trait were used for further analysis. To evaluate the effects of *Dw6* on kernel weight and size, 30 RIL lines with short stature and 30 RILs with tall plant height were randomly selected. These lines together with their parents were used to measure the hundred kernel weight and kernel size including kernel length, kernel width and kernel perimeter. Hundred kernel weight was measured by a random selection of 100 mature seeds from each line after air-dried at 35°C to constant weight, and the mean value of three replications was used. Whereas for measurement of kernel size, 30 dried seeds were randomly selected and dehulled by hand following by scanning on a flatbed scanner. The scanned images were then analyzed using the WinSEEDLE Pro 2012a Image Analysis System (Regent Instruments, Inc., Quebec, Canada) to obtain the kernel length, kernel width and kernel perimeter data.

### Assessment of Coleoptile Length at Seedling Stage

Seeds from the 60 RILs described above were used to assess the coleoptile length by using the method as described (Luo et al., 2020). Briefly, uniform, healthy-looking seeds of each line were sown at 2cm depth in individual 6 × 6 cells of germination trays filled with washed river sand (13% v/W moisture) and placed at 4°C for 2 days to remove any residual seed dormancy and ensure even germination. These trays were then stored in a growth chamber with a constant temperature of 20°C. After 14 days, the seedlings were pulled out carefully and the coleoptile length was assessed as the distance from the end of the grain to the coleoptile tip. Ten coleoptile length measurements from each line were ranked and the six longest values (free from any abnormalities) were used to calculate the mean.

### Statistical Analysis

Based on the plant height, the RILs were classified into two classes as tall (*dw6*) and dwarf (*Dw6*) in each of the three environments of testing. To avoid potential contamination in field practice, only these RILs that were uniformly grouped as dwarf or tall among all environments to be retained for further analysis. The differences in phenotype due to dwarfing gene were tested for significance by a one-tailed Student’s *t*-test. The relative effects of *Dw6* were estimated following the formula: effect = (Mean_dwarf_ - Mean_tall_)/Mean_tall_ × 100%. The Pearson correlation coefficients between plant height and other agronomic traits were estimated following with significant test by using base packages of R language.

To visualize the relationships existing between the investigated traits, a genotype by trait (GT) biplot was generated using package GGEBiplots implemented in R. GT-biplot is an implementation of the GGE-biplot technique [a methodology to graphically summarize the effects of genotype and genotype × environment interaction (Yan et al., 2000)] to study of the genotype by trait data (Yan and Rajcan, 2002). To this end, a two-way matrix of means was generated for lines and agronomic traits averaged across environments. An additional column was included in the matrix giving the grouping information (0 = dwarf or 1 = tall) for each line. A GT-biplot was built by plotting the first principal component (PC1) scores of the genotypes and the traits in relation to their corresponding scores for the second principal component (PC2) resulting from singular-value decomposition (SVD) of trait-standardized data (Yan and Rajcan, 2002). The correlation coefficient between any two traits is approximated by the cosine of the angle between their vectors (angle ≤ 90°, positive correlation; angle = 90°, no correlation; angle ≥ 90°, negative correlation), while the variations of the traits are reflected by the vector lengths (Yan and Rajcan, 2002).

### Genetic Mapping of *Dw6*

Previous studies associated an RFLP clone, aco245 with *Dw6* in seven pairs of NILs contrasting for the presence of *Dw6* (Molnar et al., 2012). However, recombinants between an SNP marker that derived from aco245 and *Dw6* have been observed, hence suggested the variation in aco245 might not be responsible for the dwarf type (Zhao et al., 2018), additional effort is necessary to further map *Dw6*. To this end, a BLASTN was performed using the sequence of aco245 (GenBank accession JF913493) as a query against the recently released hexaploid oat reference genome (*Avena sativa* – OT3098 v1, PepsiCo; see text footnote 1). Since *Dw6* has been mapped on 18D (6D in the hexaploid reference genome) chromosome (Zhao et al., 2018), therefore 1 Mb flanking sequences around 6D aco245 were extracted, which were then used for SSR identification and primer design by MISA (Thiel et al., 2003) and Primer3 (Untergasser et al., 2012), respectively. After taking into account the putative PCR production size (at least 100 bp) and chromosome location (1 marker per 20 kb), a total of 81 primer pairs (Supplementary Table 3) were used for mapping analysis. Linked SSR markers were identified using DNA bulks from 10 short and tall progenies as well as from short and tall parents. Polymorphic markers were then used to generate genotypic data for the whole RIL population and a partial genetic linkage map was generated by JoinMap version 4.0, utilizing the regression mapping algorithm to calculate marker order and the Kosambi mapping function to estimate the map distances. A LOD score of 3.0 was considered evidence for linkage. Linked markers were further validated in other oat materials as described above.

### Candidate Genes for *Dw6*

The current version (*Avena sativa*-OT3098 v1, PepsiCo) of the oat reference genome has been independently annotated by using transcripts from Hu et al. (2020) and PacBio data generated by PepsiCo, respectively (see text footnote 1). Hence, transcripts from both sources between the flanking markers were retrieved. The physical positions of some of the transcripts were overlapped, indicative of the presence of redundancy among these transcripts. Hence these transcripts were filtered based on their physical positions (i.e., the transcripts with the largest coverage were retained as the representatives). The remaining transcripts were used to search for orthologous sequences in the NCBI database^2^ using BLASTN and an E value less than e-10.

## Results

### Agronomic Comparison of Tall and Dwarf Parent

Details of phenotypic variation between two parents were summarized in Table 1. The results indicated that all investigated traits except number of whorls are significantly different between two parents in all tested environments. The tall parent Caracas displayed significantly higher values in plant height, panicle length, spikelet number per panicle, hundred kernel weight, kernel width, kernel length, kernel perimeter as well as coleoptile length than the dwarf line WAOAT2132, and it also showed more whorls per panicle compared to the latter (Table 1). However, the productive tiller number of the dwarf parent WAOAT2132 was significantly less than that of the tall parent.

**TABLE 1:** The mean value along with standard deviation of plant height and other investigated agronomic traits observed in *Dw6* containing parent WAOAT2132 and tall parent Caracas in different growth environments.

**Trait^a^**	**Environment^b^**	**Mean ± SD**		**Difference^c^**	**Percentage (%)^d^**
		**P1 (WAOAT2132)**	**P2 (Caracas)**		
PH (cm)	2018–2019 (WJ)	96.26.3	167.92.8	−71.7**	–42.7
	2018–2019 (CZ)	78.86.7	134.57.8	−55.7**	–41.41
	2019–2020 (WJ)	90.54.4	166.33.2	−75.8**	–45.58
PL (cm)	2018–2019 (WJ)	25.22.2	34.41.7	−9.2**	–26.74
	2018–2019 (CZ)	22.44.7	28.15.8	−5.7**	–20.29
	2019–2020 (WJ)	22.31.1	31.44.7	−9.1**	–28.98
NW	2018–2019 (WJ)	7.60.5	8.40.9	–0.8	–9.52
	2018–2019 (CZ)	8.80.8	9.40.5	–0.6	–6.38
	2019–2020 (WJ)	8.60.5	9.01.2	–0.4	–4.44
SP	2018–2019 (WJ)	52.07.3	72.06.7	−20.0**	–27.78
	2018–2019 (CZ)	70.613.2	97.015.2	−26.4*	–27.22
	2019–2020 (WJ)	73.36.6	98.310.0	−25.0**	–25.43
PTN	2018–2019 (WJ)	14.51.3	11.81.9	2.7*	22.88
	2018–2019 (CZ)	11.02.5	8.01.0	3.0*	37.50
HKW (g)	2018–2019 (WJ)	3.60.08	4.40.01	−0.8**	–18.18
	2018–2019 (CZ)	3.20.10	4.30.11	−1.1**	–25.58
	2019–2020 (WJ)	2.90.17	4.00.19	−1.1**	–27.50
KL (mm)	2018–2019 (WJ)	8.80.72	9.30.42	−0.5**	–5.38
	2018–2019 (CZ)	8.80.36	9.80.73	−1.0**	–10.20
	2019–2020 (WJ)	7.90.49	9.80.67	−1.9**	–19.39
KW (mm)	2018–2019 (WJ)	2.40.23	3.00.09	−0.6**	–20.00
	2018–2019 (CZ)	2.60.13	2.80.24	−0.15**	–5.45
	2019–2020 (WJ)	2.60.20	2.80.17	−0.20**	–7.14
KP (mm)	2018–2019(WJ)	22.21.84	24.41.38	−2.2**	–9.02
	2018–2019 (CZ)	21.31.03	25.12.36	−3.8**	–15.14
	2019–2020 (WJ)	19.91.50	25.62.27	−5.7**	–22.27
CL (cm)	2018–2019 (WJ)	10.21.10	12.40.31	−2.2**	–17.74
	2018–2019 (CZ)	9.91.08	11.41.72	−1.5*	–13.16

^*a*^PH, plant height; PL, panicle length; NW, number of whorls on the main tiller; SP, spikelet number per panicle; PTN, productive tiller number; HKW, hundred kernel weight; KL, kernel length; KW, kernel width; KP, kernel perimeter; CL, coleoptile length. ^*b*^WJ, Wenjiang; CZ, Chongzhou. ^*c*^Difference was calculated by the mean value of the dwarf parent minus that of the tall parent, and tested for significance using one-tailed Student’s t-test (*, significant at *p* < 0.05; **, significant at *p* < 0.01). ^*d*^The percentage was estimated by percentage of the difference to tall parent.

### The Effects of *Dw6* on Agronomic Traits in RIL Lines

**FIGURE 1:**
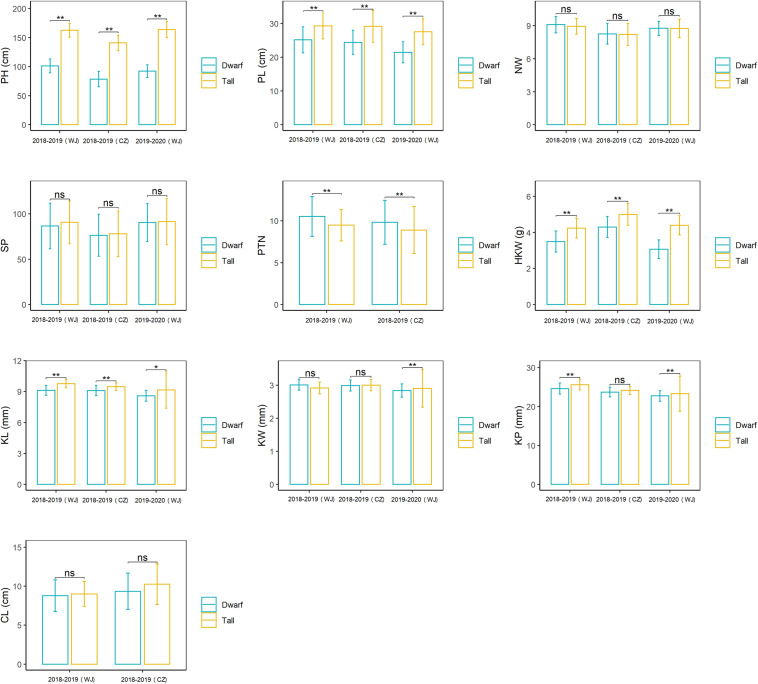
The effects of *Dw6* on plant height (PH), panicle length (PL), Number of whorls on the main tiller (NW), spikelet number per panicle (SP), productive tiller number (PTN), hundred kernel weight (HKW), kernel length (KL), kernel width (KW), kernel perimeter (KP) and coleoptile length (CL). Significance between tall and dwarf groups was tested using a one-tailed Student *t*-test. ns, non-significant; *, significant at *p* < 0.05; **, significant at *p* < 0.01.

The RIL population showed a bimodal distribution for plant height in each of the three environments of testing (Supplementary Figure 1), confirming the height of this population was governed by a single major gene. RILs in this population were subsequently divided into two groups, tall vs dwarf, on the basis of the plant height. Most of them (264/269) were consistent in categorization in all environments and used to evaluate the effects of *Dw6* on agronomic traits (Supplementary Table 1). The ranges, mean values along with standard deviation (SD) for the tall and dwarf groups were summarized in Figure 1, the mean differences between these two groups and the effects of dwarfing gene on agronomic traits were estimated and were given in Table 2 (see Supplementary Table 1 for raw data). The plant height and panicle length of the dwarf group were significantly shorter by at least 37.66 and 13.99%, respectively, than that of the tall group (Table 2), indicating a very strong negative association between *Dw6* and plant height, as well as between *Dw6* and panicle length. However, such reduction in plant height and panicle length between the tall and dwarf groups in the RIL population was less than that in the two parents. A strong negative effect of *Dw6* on kernel weight was also observed (Table 2), which could cause an average reduction of 20.07% for kernel weight across three environments. Further comparisons of the kernel sizes revealed that the kernel length of the dwarf group is significantly shorter than that of the tall group in each of the three environments of testing (Table 2). These results indicated a strong negative association between *Dw6* and kernel length. Correspondingly, the kernel perimeter of the dwarf group was less than that of the tall group by 3.79% in general (Table 2). Unlike the negative effects of *Dw6* on kernel length, the effects of *Dw6* on kernel width were inconsistent in three environments (Table 2), which suggested that kernel width might be affected more by the environment, rather *Dw6* in this study. By contrast, lines with *Dw6* have more productive tillers than *Dw6*-absence lines by 10.53 and 10.11% in 2018–2019 (WJ) and 2018–2019 (CZ), respectively, indicative of a significant positive association between *Dw6* and productive tiller number. Number of whorls per panicle was the same for lines containing tall and dwarf *Dw6* alleles. Interestingly, the spikelet number per panicle, a closely yield-related trait that was much lower (26.81% on average) in the dwarf parent than that in the tall parent, showed no significant difference between tall and dwarf lines, suggesting a weak association between *Dw6* and this trait.

**TABLE 2:** Ranges, means, and differences for investigated agronomic traits between tall and dwarf F_6:8_ lines of WAOAT2132/Caracas population and the estimated effects of *Dw6*.

**Trait**	**Environment**	**Tall (*dw6*)**		**Dwarf (*Dw6*)**		**Difference^a^**	**Effect (%)^b^**
		**Range**	**Mean**	**Range**	**Mean**		
PH (cm)	2018–2019(WJ)	132.4–194.7	162.512.0	70.2–126.1	101.312.1	−61.2**	–37.66
	2018–2019(CZ)	110.6–184.4	140.913.3	46.4–106.4	78.513.4	−62.4**	–44.29
	2019–2020(WJ)	134.4–198.4	163.813.9	63.8–113.6	92.110.8	−71.7**	–43.77
PL (cm)	2018–2019(WJ)	21.0–40.0	29.33.9	16.3–37.0	25.23.9	−4.1**	–13.99
	2018–2019(CZ)	17.5–39.5	29.24.7	14.4–32.2	24.43.6	−4.8**	–16.44
	2019–2020(WJ)	19.9–38.1	27.63.8	14.8–35.5	21.53.1	−6.1**	–22.10
NW	2018–2019(WJ)	7.6–10.8	8.90.7	6.6–11.0	9.10.8	0.2	2.25
	2018–2019(CZ)	5.6–10.4	8.21.0	5.6–9.8	8.30.9	0.1	1.22
	2019–2020(WJ)	7.2–11.0	8.80.8	7.2–10.8	8.80.6	0.0	0.00
SP	2018–2019(WJ)	45.0–149.6	90.623.7	42.6–169.4	86.525.2	–4.1	–4.53
	2018–2019(CZ)	32.0–137.6	78.025.2	30.6–145.4	76.323.1	–1.7	–2.18
	2019–2020(WJ)	42.2–165.8	91.525.4	54.6–153.8	90.520.9	–1.0	–1.09
PTN	2018–2019(WJ)	4.8–15.8	9.51.9	3.8–16.4	10.52.4	1.0**	10.53
	2018–2019(CZ)	4.2–17.8	8.92.8	4.6–16.2	9.82.6	0.9**	10.11
HKW (g)	2018–2019(WJ)	3.3–5.4	4.20.6	2.7–4.7	3.50.6	−0.7**	–16.67
	2018–2019(CZ)	3.6–6.6	5.00.6	3.4–5.2	4.30.6	−0.7**	–14.00
	2019–2020(WJ)	2.8–5.2	4.40.5	2.0–3.9	3.10.5	−1.3**	–29.55
KL (mm)	2018–2019(WJ)	9.1–10.5	9.80.40	8.1–10.3	9.10.48	−0.7**	–7.14
	2018–2019(CZ)	8.8–10.2	9.50.4	8.2–10.1	9.10.5	−0.4**	–4.21
	2019–2020(WJ)	8.7–10.1	9.50.4	8.7–9.8	8.60.5	−0.9*	–9.47
KW (mm)	2018–2019(WJ)	2.5–3.3	2.90.2	2.6–3.3	3.00.2	0.1	3.45
	2018–2019(CZ)	2.6–3.3	3.00.2	2.6–3.3	3.00.2	0.0	0.00
	2019–2020(WJ)	2.8–3.3	3.00.1	2.3–3.1	2.80.2	−0.2**	–6.67
KP (mm)	2018–2019(WJ)	23.1–28.0	25.61.3	22.6–28.7	24.61.4	−1.0**	–3.91
	2018–2019(CZ)	22.5–26.2	24.11.0	21.3–26.1	23.71.3	–0.4	–1.66
	2019–2020(WJ)	21.7–25.8	24.11.1	20.5–25.8	22.71.4	−1.4**	–5.81
CL (cm)	2018–2019(WJ)	5.9–12.4	9.01.6	4.5–12.9	8.82.1	–0.2	–2.22
	2018–2019(CZ)	6.3–17.3	10.32.6	4.8–14.6	9.32.3	–1.0	–9.71

^*a*^Difference was calculated by the mean value of dwarf group minus that of the tall group, and tested for significance using one-tailed Student’s *t*-test (*, significant at *p* < 0.05; **, significant at *p* < 0.01). ^*b*^The effect was estimated by following the formula: Effect = (Mean_*dwarf*_ - Mean_*tall*_)/Mean_*tall*_ × 100%.

Correlation coefficients between plant height and other agronomic traits in the RIL lines were shown in Table 3, which revealed that plant height was very positively and significantly correlated with panicle length (*r* = 0.69), whereas it had a negative but not significant correlation with productive tiller number. Highly significant correlations were also observed between plant height and hundred kernel weight (*r* = 0.57), kernel width (*r* = 0.53), and kernel perimeter (*r* = 0.37). No significant correlation was observed between plant height and number of whorls, spikelet number per panicle, kernel length or coleoptile length.

**TABLE 3:** Pearson correlation coefficients between plant height and other agronomic traits in the F_6:8_ RIL lines of WAOAT2132/Caracas.

**Traits**	**F_6:8_ RILs**
PL	0.69**
NW	0.05
SP	0.16
PTN	–0.21
HKW	0.57**
KW	0.14
KL	0.53**
KP	0.37**
CL	0.10

**FIGURE 2:**
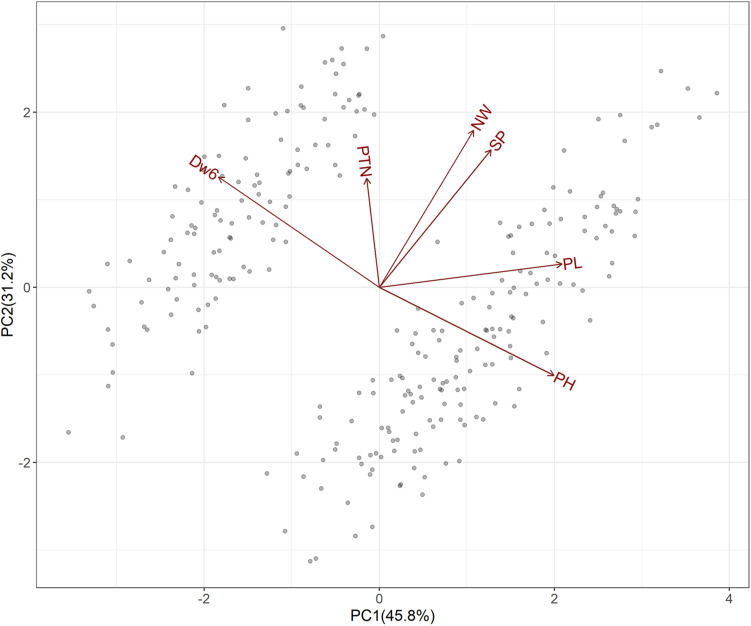
Biplot following principal components analysis of F_6:8_ RIL lines from the WAOAT2132/Caracas population varying for *Dw6* alleles.

Presence of *Dw6* and subsequent effects on plant height and some other agronomic traits including panicle length, number of whorls, productive tiller number and spikelet number per panicle was plotted in Supplementary Figure 2 and also examined with PCA and the fundamental patterns among the traits were illustrated by a GT-biplot (Figure 2). The GT-biplot explained 77% of the total variation of the standardized data. The large, obtuse angle between vectors defining plant height and the *Dw6* allele indicated a strong negative correlation for these two traits. A strong negative correlation between *Dw6* and panicle length was also revealed by the large obtuse angle. The near perpendicular vectors indicated a near-zero correlation between *Dw6* and number of whorls, as well as between *Dw6* and spikelet number per panicle, whereas the 45° angle between vectors defining *Dw6* and productive tiller number suggested a positive correlation for these two traits. Other prominent relations revealed from the GT-biplot included a strong positive correlation between number of whorls and spikelet number per panicle and independent variation between panicle length and productive tiller number. The relations among agronomic traits revealed by the GT-biplot were well matched with correlation coefficients as shown in Table 3, indicating the usefulness of GT-biplot in graphically summarizing relationships among agronomic traits.

### Mapping *Dw6* in the RIL Population

**FIGURE 3:**
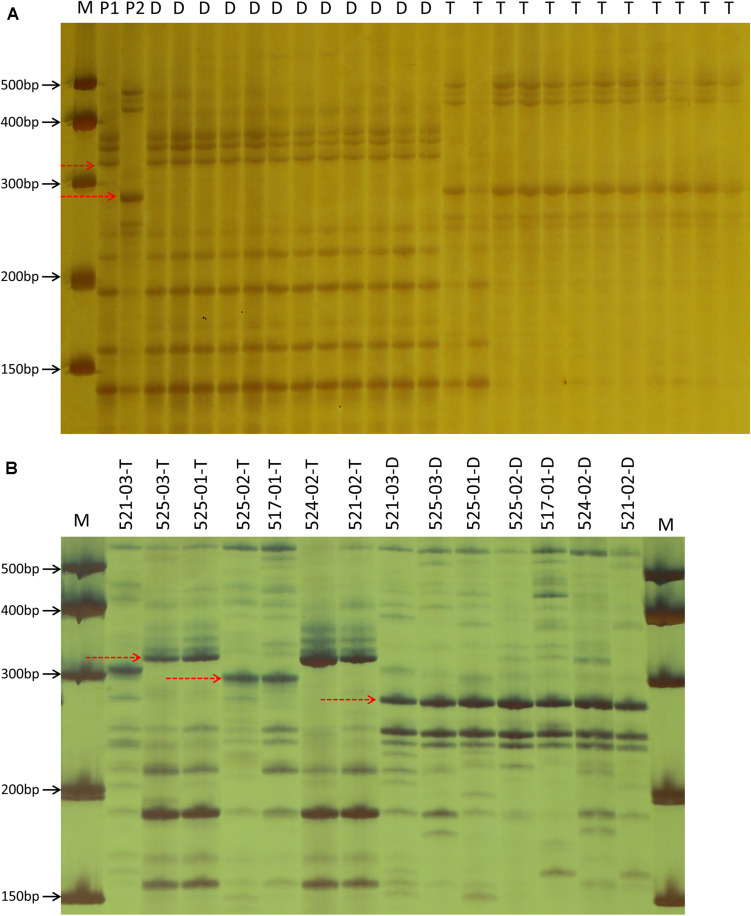
Example of marker profile generated by the primer pair SSR120 in two parents and partial F_6:8_ progenies **(A)**, as well as in 14 near-isogenic lines contrasting for *Dw6* **(B)**. M: marker, P1: WAOAT2132, P2: Caracas, D: dwarf progeny, T: tall progeny. The dotted arrow indicates the polymorphic bands in this locus. Character after underscore line in part **(B)** represents the phenotype of corresponding material, T, tall; D, dwarf.

**FIGURE 4:**
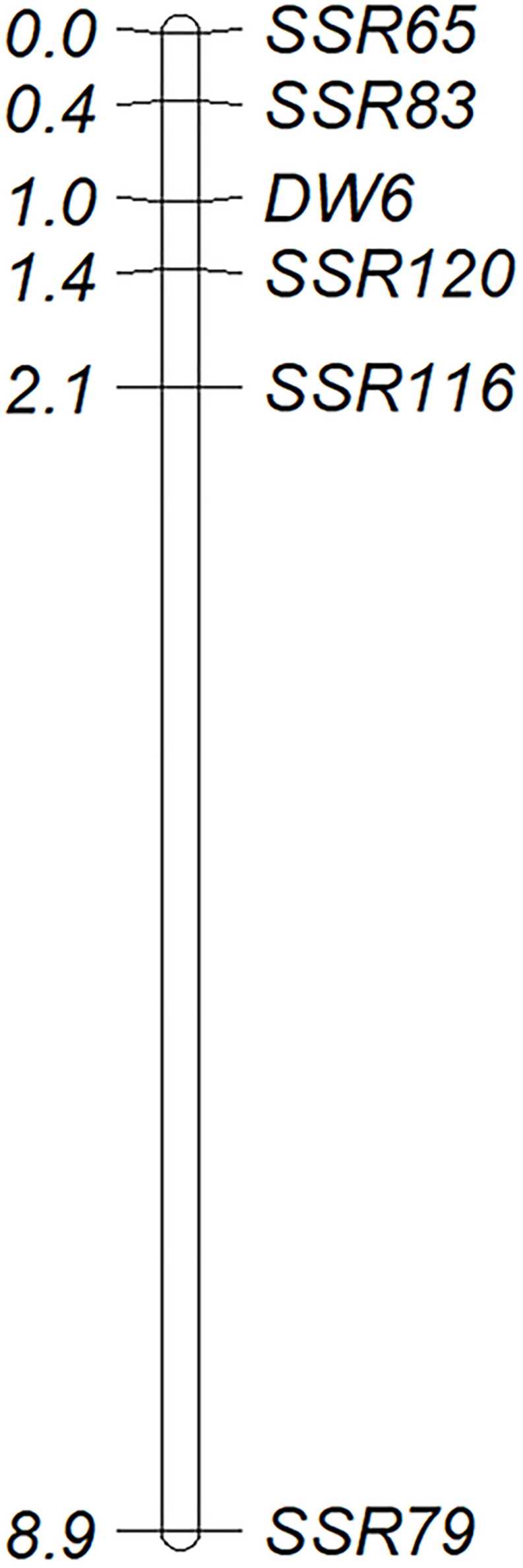
A partial genetic map of *Dw6* based on data from 264 F_6:8_ families of WAOAT2132 × Caracas population. Genetic distances in centiMorgans (cM) are given on the **left** and marker loci are named on the **right** side of the map (LOD = 3.0).

The BLASTN analysis by using the aco245 sequence as a query against the recently released hexaploid oat reference genome identified three sequences located on 6D, 6A, and 6C to be highly similar with aco245. Because *Dw6* has been mapped on the D chromosome (Zhao et al., 2018), hence one Mb sequences flanking the sequence on 6D were extracted and used for SSR marker identification. A total of 81 SSR primer pairs were used (Supplementary Table 3) to test for polymorphism in the F_6__:__8_ RIL population. Of these, five pairs showed polymorphism both in parents and in the contrasting bulks. These polymorphic SSR markers were used to genotype the whole RIL population (Supplementary Table 1 and Figure 3A), and then the generated genotype data were used to create a partial genetic map where the plant height of the RILs was scored as a binary trait and mapped as a genetic marker. *Dw6* was mapped to an interval of 1.0 cM flanked by markers SSR83 and SSR120 (Figure 4). The linkage between *Dw6* with SSR120 was further validated in seven pairs of NILs contrasting for *Dw6*. Polymorphisms were observed for SSR120 in the 14 NILs, which corresponded exactly with their plant heights (Figure 3B and Supplementary Table 2). The allelic frequency of the most linked marker SSR120 was tested on a set of 48 diverse oat accessions including ten hulless oats and 38 hulled oats (three accessions OT207, Potoroo and AC Ronald have been reported to carry *Dw6*). The result showed that marker SSR120 differentiated all semi-dwarf lines including these *Dw6*-containing accessions and one dwarf line Drummond with an unknown dwarf gene from the others (Supplementary Figure 3 and Table 2).

### Candidate Gene

A total of 12 and 5 transcripts from Hu et al. (2020) and PacBio data generated by PepsiCo were found within the region where *Dw6* was mapped, respectively. After de-redundancy, nine transcripts were retained and BLASTN analyzed. Of them, six showed high homology to wheat, *Aegilops tauschii*, and other plant sequences annotated as auxin-responsive protein SAUR32, histone-lysine *N*-methyl transferase SUVR4-like, pentatricopeptide repeat-containing protein, *Avena* OP45 receptor kinase, MIS2 protein, and E3 ubiquitin-protein ligase WAV3, respectively (Table 4).

**TABLE 4:** Detailed information on predicted genes within the region where *Dw6* was mapped in this study.

**Gene ID**	**Physical position (bp)**	**Function**
TRINITY_DN32248_c0_g1_i1.path1	244773360-244773673	*Aegilops tauschii* subsp. *strangulata* auxin-responsive protein SAUR32 (LOC109753154)
Pepsico1_Contig31797.path3	244858840-244862325	*Triticum dicoccoides* histone-lysine N-methyltransferase SUVR4-like (LOC119352070)
TRINITY_DN87243_c0_g1_i1.path1	244876840-244877097	NA
TRINITY_DN34795_c0_g1_i1.path1	244881344-244882291	*Aegilops tauschii* subsp. *strangulata* pentatricopeptide repeat-containing protein At1g33350 (LOC109745475)
TRINITY_DN26579_c0_g1_i2.path3	244907567-244912581	*Avena sativa* clone OP45 receptor kinase gene cluster
Pepsico2_Contig12323.path1	244945718-244946577	NA
Pepsico2_Contig4854.path2	244947664-244952796	*Aegilops tauschii* subsp. *strangulata* protein MIS12 homolog (LOC109776041)
TRINITY_DN77282_c0_g1_i1.path2	245196577-245203214	NA
Pepsico2_Contig3963.path1	245313093-245318882	*Aegilops tauschii* subsp. *strangulata* E3 ubiquitin-protein ligase WAV3 (LOC109774683)

*A BLASTN search against the NCBI database (http://blast.ncbi.nlm.nih.gov/Blast.cgi) was performed to search for orthologous sequences with an E value less than e-10. NA: No significant similarity was found.

## Discussion

### The Effects of *Dw6* on Agronomic Traits

This study was carried out to get a better understanding of the genetic effects of the GA-sensitive *Dw6*, the only dwarfing gene in oat that has been used for cultivar development, on a range of agronomic traits, including plant height, coleoptile length and yield-related traits. Homozygous F_6__:__8_ lines derived from a cross between WAOAT2132/Caracas were used in this study to evaluate the effects of *Dw6* in a subtropical environment in southwest China.

Large and repeatable differences were observed for plant height in the WAOAT2132/Caracas population varying for the *Dw6* allele. This morphological variation could be largely attributed to allelic variation at *Dw6* loci (Figure 1 and Table 2). That is, the presence of *Dw6* was closely related to reductions in plant height ranging from 37.66 to 44.29% across three environments of testing. The large height reduction associated with *Dw6* was consistent with the height reductions reported for *Dw6* in Kibite (2001) and Milach et al. (2002). Likewise, there were large and repeatable differences in the panicle lengths among lines in the WAOAT2132/Caracas population. The variation in panicle length was strongly correlated (*r* = 0.69, *P* < 0.01) with plant height, indicating the panicle length to be an important determinant of overall stature. Lines with *Dw6* had reduced panicle length by 17.51% on average compared with tall lines. This was well in accordance with the previous study (Milach et al., 2002).

A small amount of work has been conducted to evaluate the effects of *Dw6* on yield and yield components (Brown et al., 1980; Barr, 1984; Marshall et al., 1987; Anderson and McLean, 1989; Kibite and Clayton, 2000), however, no consistent results have been observed. Brown et al. (1980) reported the dwarf line produced similar yields to standard-height cultivars at five locations in the eastern Canadian prairies. In Australia, *Dw6*-containing cultivar Echidna outyielded most of the formerly widely grown tall cultivars, especially in the south-western, high rainfall areas. However, both Marshall et al. (1987) and Kibite and Clayton (2000) observed a strong negative association between *Dw6* and yield. In this study, the genetic effects of *Dw6* on three yield-related traits, including productive tiller number, spikelet number per panicle and hundred kernel weight were evaluated. *Dw6* has no significant effect on spikelet number, but is strongly negatively associated with the reduction of kernel weight (Table 2 and 3). The average hundred kernel weight of 30 RIL lines with tall plant height across three environments was 4.5 g, which was 20.07% lower than that of RILs with short stature (Figure 1 and Table 2). Further comparing the kernel sizes of these two groups revealed that the kernel lengths of dwarf lines are significantly shorter than that of tall lines (8.9 *vs* 9.6 mm on average across three environments), whereas the kernel widths of these lines are similar (Figure 1 and Table 2). These results were in line with previous studies (Brown et al., 1980; Kibite and Clayton, 2000), which also observed a negative effect by *Dw6* on kernel size. All these results together suggested that the potential yield penalty associated with *Dw6* should be partially caused by the reduction of kernel weight which is largely attributed to the decrease of kernel length. Indeed, most of the reported dwarfing genes in wheat like *Rht1* (McClung et al., 1986), *Rht13* (Rebetzke et al., 2011), *Rht18* (Yang et al., 2015), *Rht14* (Duan et al., 2020) and *Rht15* (Zhao et al., 2021) have negative effects on grain weight and grain size, suggesting a common defect of dwarf genes. This might not be unexpected because most dwarfing genes reduced the plant height via blocking the biosynthesis or utilization of phytohormones, predominantly gibberellins (Liu et al., 2018), which were also required for grain development. Besides, the flowering date of the *Dw6* dwarf lines was 3–7 days later than that of the tall lines (data not shown) as observed by a previous study (Kibite and Clayton, 2000), which might be another reason for the small kernels as the grain-filling stage was reduced (Duan et al., 2020; Zhao et al., 2021). The small grain associated with *Dw6* might be improved by combining with the genes regulating grain-filling (Duan et al., 2020; Zhao et al., 2021). Moreover, the yield penalty caused by the small kernel size associated with dwarfing gene might be compensated for by increases of other yield components. In turn, harvest index and grain yields of these dwarf genotypes are similar with or even higher than that of the tall genotypes. For instance, wheat cultivars with *Rht1* produced much smaller kernels compared to the conventional tall wheats, but this was completely compensated for by a greater number of tillers and kernels per spike (McClung et al., 1986). In this study, RIL lines with *Dw6* had a significantly higher number of productive tillers than that of the tall lines. This attribute of *Dw6* would partially attenuate the negative effects of *Dw6* on yields, but further work is required to confirm this since the yields were not evaluated in this study.

Because most dwarfing genes are associated with the reduction of cell length in the peduncle, they might also affect the length of other organs. Coleoptile is a sheath-like tissue that protects the emerging shoot and delivers it to the soil surface (Luo et al., 2020). It plays an important role in early crop establishment and its length determines the maximum depth at which seed can be sown (Sidhu et al., 2020). Considerable studies have been conducted to evaluate the relations between wheat dwarfing genes and coleoptile length (Ellis et al., 2004; Rebetzke et al., 2012; Chen et al., 2013; Tang, 2016). The results indicated that the GA-insensitive gene *Rht1* is strongly positively related to the short coleoptile (Rebetzke et al., 2012), whereas most GA-sensitive dwarf genes have no significant effects on coleoptile length (Rebetzke et al., 2012; Chen et al., 2013; Tang, 2016). In this study, the dwarf parent showed a significantly shorter coleoptile compared with that of the tall parent (Table 1), however, this large difference had not been observed between the tall and dwarf groups of the F_6__:__8_ RILs though the coleoptile lengths of the tall lines are longer than that of the dwarf lines (Figure 1 and Table 2). These results indicated that coleoptile length is genetically independent of the height reduction caused by *Dw6*, thus breeding oat cultivars with a combination of long coleoptile and reduced plant height (*Dw6*) is practicable.

### Implications for Use of *Dw6* for Oat Cultivar Development

Despite the negative effects of *Dw6* on grain yield or yield components, it may have great potential in oat breeding for the following reasons. First, the effectiveness of *Dw6* in reducing oat plant height was observed in all studies. Second, great variations were observed in both tall and dwarf groups in this study (Table 2 and Figure 1), which provided great potential for selecting individuals with proper height for oat production (i.e., choose relative higher individuals without lodging). Third, *Dw6* had a significant positive effect on productive tiller number (Figure 2 and Table 3). Hence, the potential yield penalty would be compensated for by an increase of panicle number per unit. Indeed, previous studies have demonstrated that an increase in seeding rate and reduction of row spacing would largely increase the grain yields of dwarf cultivars, making both dwarf and tall cultivars having similar harvest indexes (Meyers et al., 1985; Marshall et al., 1987). Besides, no significant effect of *Dw6* on coleoptile length has been observed in this study. This indicated *Dw6* may be useful also in regions with less rainfall, under which conditions, deep sowing is often necessary. However, a previous study reported QTL for Fusarium head blight (FHB) resistance were overlapped with the QTL region for *Dw6*, suggesting a negative association between *Dw6* and FHB resistance (Stancic, 2016). Another study also observed cultivar with *Dw6* is more susceptible to FHB (Herrmann et al., 2020). Whether the increased FHB susceptibility associated with the *Dw6* is attributed to genes linked to FHB susceptibility or/and a pleiotropic effect of *Dw6* allele enhancing susceptibility are not clear. This needs further studies to clarify and then provide better guidance on the utilization of *Dw6* in oat breeding.

### Genetic Mapping of *Dw6* and Developing PCR-Based Markers for *Dw6*

Attempts have been made to genetically map *Dw6* by using various types of molecular markers (Milach et al., 1997; Tanhuanpää et al., 2006; Molnar et al., 2012; Zhao et al., 2018). The initial study mapped *Dw6* using RFLP markers, which identified one unmapped RFLP marker Xumn145B to be putatively linked to *Dw6*. Further analysis revealed this locus was absent in oat lines missing chromosome 18, thus located *Dw6* on chromosome 18 (Milach et al., 1997). Tanhuanpää et al. (2006) identified two SNP markers that were closely linked to *Dw6*, which were located 5.2 and 12.6 cM from *Dw6*, but their locations on a reference map were not determined. Later on, Molnar et al. (2012) identified an RFLP marker aco245 to be closely linked to *Dw6* by using a set of NILs for *Dw6*. This RFLP marker was revealed to be located on the KO LG_33 linkage group, which is homologous to chromosome 18D (Oliver et al., 2013) and linkage group Mrg04 of the most recent consensus map (Chaffin et al., 2016). Mrg04 has been found to be homologous to chromosome 6D of the recently released hexaploid oat reference genome (data not shown). In this study, the aco245 sequence was blasted against the recently released hexaploid oat reference genome, which identified three highly similar sequences located on 6D, 6A and 6C, respectively. Five SSR primer pairs around the 6D aco245 sequence showed polymorphism in two parents and in two contrasting bulks. These results confirmed that *Dw6* is located on chromosome 6D. By using these five polymorphic SSR markers, *Dw6* was mapped at an interval of 1.0 cM flanked by markers SSR83 and SSR120. Caution should be applied in using this information since the genetic orders of the five polymorphic markers on the genetic map in this study were not fully consistent with their physical locations on the reference genome (Figure 4). Such inconsistencies may reflect the actual structural differences caused by, for example, chromosomal segmental rearrangements, segmental duplications, between the reference genome and the parental lines used in this study. However, the discrepancies might also be addressed by assembling errors, which often happened, particularly in genome assemblies of the large, highly repetitive genome as oat (Liu et al., 2019). Previous studies have identified some markers to be putatively linked to *Dw6*, however, none of them are perfectly diagnostic for marker-assisted selection (MAS). For example, the SCAR markers developed by Molnar et al. (2012) were monomorphic on some NILs of testing. SSR marker bi17 was assumed to be linked to *Dw6*, however, fragments representing the dwarf allele existed in tall NILs as well as in some tall cultivars (Zhao et al., 2018). In this study, SSR120 was revealed to be the closest marker linked to *Dw6*. To validate the accuracy of these markers in the identification of *Dw6*, the polymorphism of SSR120 was examined in a range of oat lines including seven pairs of NILs with different genetic backgrounds and 48 diverse cultivars. The results indicated that marker SSR120 accurately discriminated the lines with *Dw6* from the others (Supplementary Figure 3), demonstrating their usefulness in the rapid identification of *Dw6*.

### Candidate Gene for *Dw6*

A previous study suggested the gene corresponding to the cDNA clone aco245 and coding for V-ATPase subunit H as the candidate gene for *Dw6* locus since the multimeric V-ATPase enzymes were functionally conserved in plants and known to play a key role in regulation of cell elongation and plant growth (Obroucheva, 2008; Sharma et al., 2009). In this study, the gene corresponding to 6D aco245 was ∼80 and ∼700 Kb distant from marker SSR65 and SSR120, respectively. Besides, based on the reference genome sequence, sequences of the open reading frame of this gene in both tall and dwarf parents were cloned but showed 100% identity with each other (data not shown). All these results together may suggest that variation of the gene corresponding to 6D aco245 should not be responsible for the dwarf phenotype.

A preliminary annotation has been performed based on transcripts from Hu et al. (2020) and PacBio data generated by PepsiCo for the version 1 OT3098 reference genome assembly. A total of nine transcripts with non-redundancy were identified within the region where *Dw6* was mapped in this study. One (TRINITY_DN32248_c0_g1_i1.path1) of them was found to have high homology to wheat and other plant sequences annotated as auxin-responsive protein SAUR32 (Table 4), a potential candidate gene for the *Dw6/dw6* locus. SAURs (Small auxin-up RNAs) are the early auxin-responsive genes represented by a large multigene family in plants (Hagen and Guilfoyle, 2002). Many studies have reported the wide involvement of SAURs in regulation of plant growth, particularly cell elongation, via auxin signaling, as reviewed by Stortenbeker and Bemer (2019), hence this gene might be considered as the potential candidate gene for *Dw6* locus. A previous study revealed that line with *Dw6* is responsive to exogenously added GA (Milach et al., 2002), but no such study has been performed to test the response of *Dw6* to auxin. However, much evidence has been reported the crosstalk between GA and other phytohormones functions in plant height control, as reviewed by Wang et al. (2017). Besides, there is one gene (Pepsico2_Contig3963.path1) that showed high homology to sequences annotated as E3 ubiquitin-protein ligase, which belongs to a protein family that is well known to control every aspect of eukaryotic by promoting protein ubiquitination and degradation (Zheng and Shabek, 2017). In barley, the dwarf gene *Brh2* encodes a U-box E3 ubiquitin ligase, *brh2* mutants showed a strong semi-dwarf phenotype (Braumann et al., 2018). Likewise, a spontaneous rice mutant, *erect leaf1*, a gene encodes a U-box protein which possesses E3 ubiquitin ligase activity, produced a dwarf phenotype with short grains (Sakamoto et al., 2013). Caution is also advisable when drawing conclusions because the current annotation is relatively preliminary which might underestimate the actual number of genes in this region. Therefore, additional efforts and a fully annotated reference genome are needed to identify the causal gene for *Dw6*.

## Data Availability Statement

The original contributions presented in the study are included in the article/Supplementary Material, further inquiries can be directed to the corresponding author/s.

## Author Contributions

YP and HY conceived and designed the experiments and wrote the manuscript. KY, PZ, JZ, YX, YL, and XL performed the field experiments. HY and KY analyzed data. HY, CR, and YP revised the manuscript. All authors read and approved the final manuscript.

## Conflict of Interest

The authors declare that the research was conducted in the absence of any commercial or financial relationships that could be construed as a potential conflict of interest.

## Footnotes

1 https://wheat.pw.usda.gov/GG3/graingenes_downloads/oat-ot3098-pepsico

2 http://blast.ncbi.nlm.nih.gov
