# Supplementary figures and images for: Position Validation of the Dwarfing Gene Dw6 in Oat (Avena sativa L.) and Its Correlated Effects on Agronomic Traits
Source: Front Plant Sci. 2021 May 20;12:668847. doi: 10.3389/fpls.2021.668847 (PMC8172587; doi:10.3389/fpls.2021.668847)

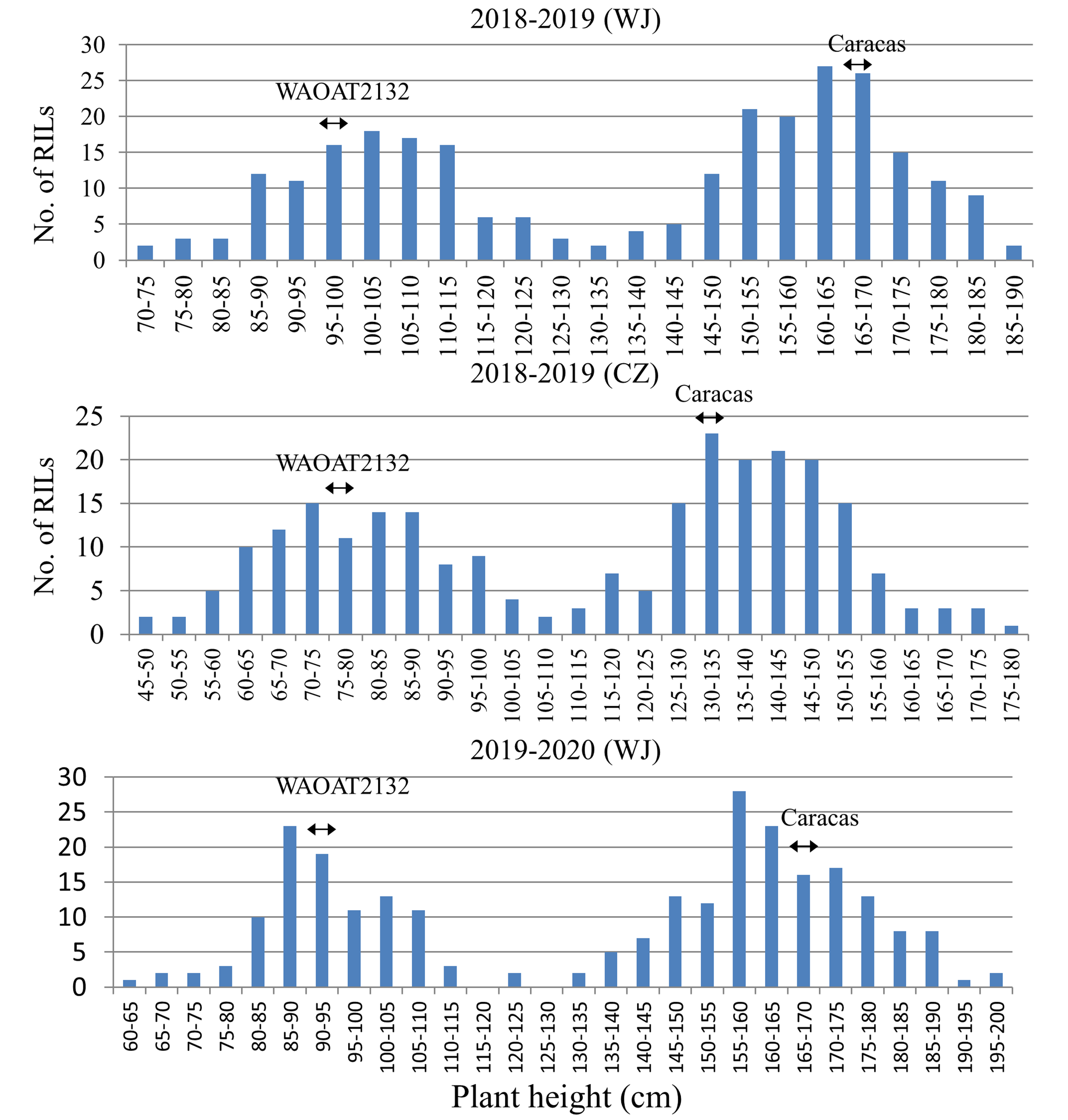

Supplement: Supplementary file 1 [file Image_1.TIF]

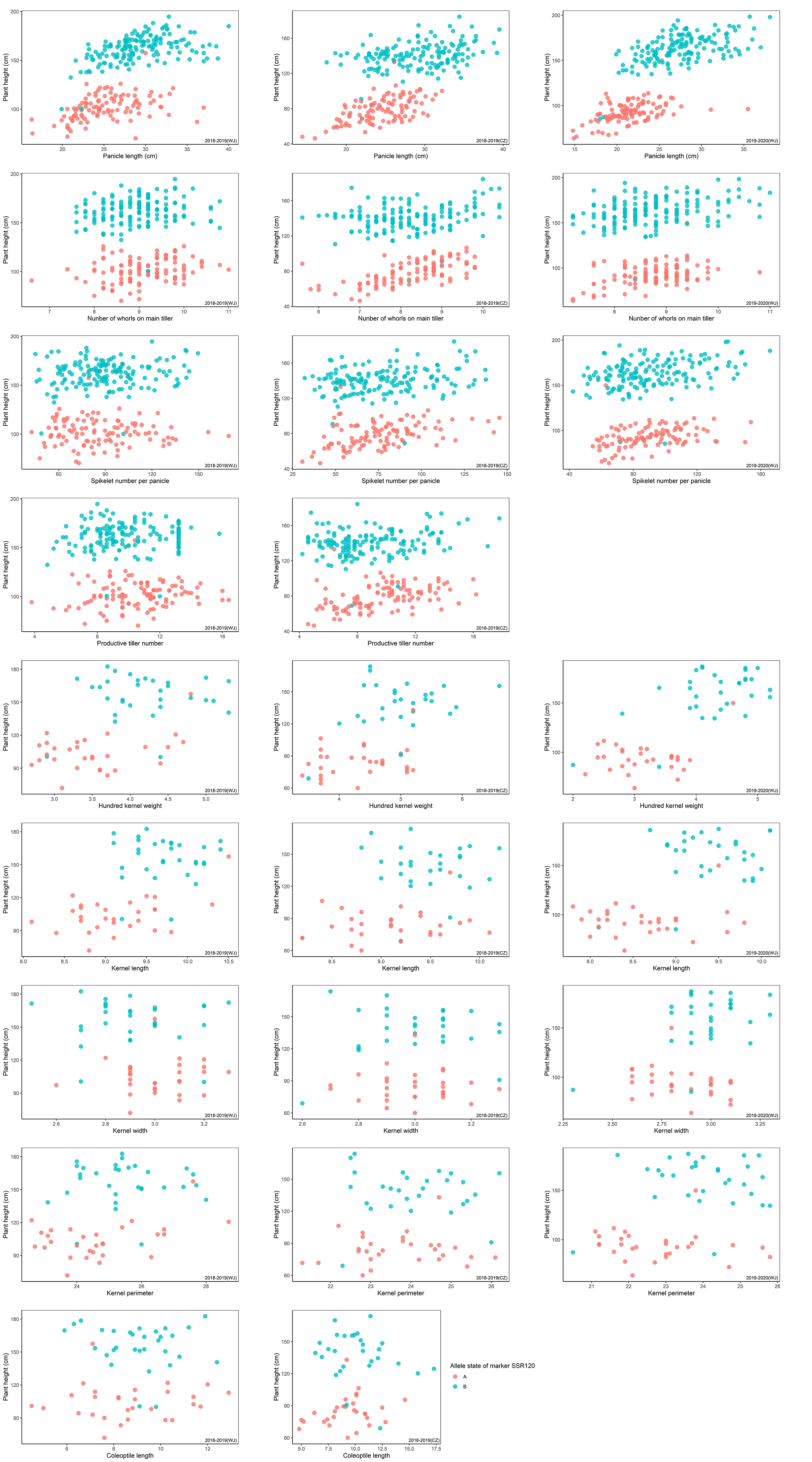

Supplement: Supplementary file 2 [file Image_2.TIF]

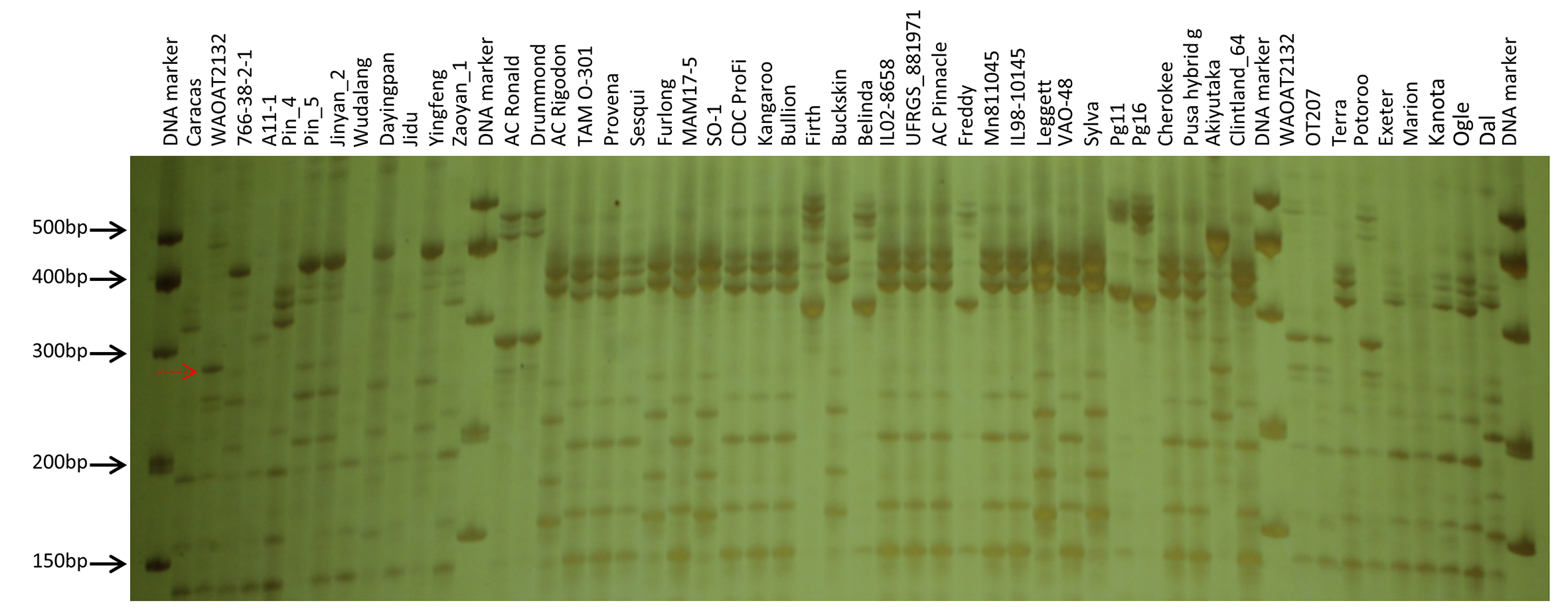

Supplement: Supplementary file 3 [file Image_3.TIF]
