# Supplementary material for: Position Validation of the Dwarfing Gene Dw6 in Oat (Avena sativa L.) and Its Correlated Effects on Agronomic Traits
Source: Front Plant Sci. 2021 May 20;12:668847. doi: 10.3389/fpls.2021.668847 (PMC8172587; doi:10.3389/fpls.2021.668847)
Supplement: Supplementary file 4 [file Data_Sheet_1.docx]

Supplementary Material

# Supplementary Data

# Supplementary Figures and Tables

## Supplementary Figures


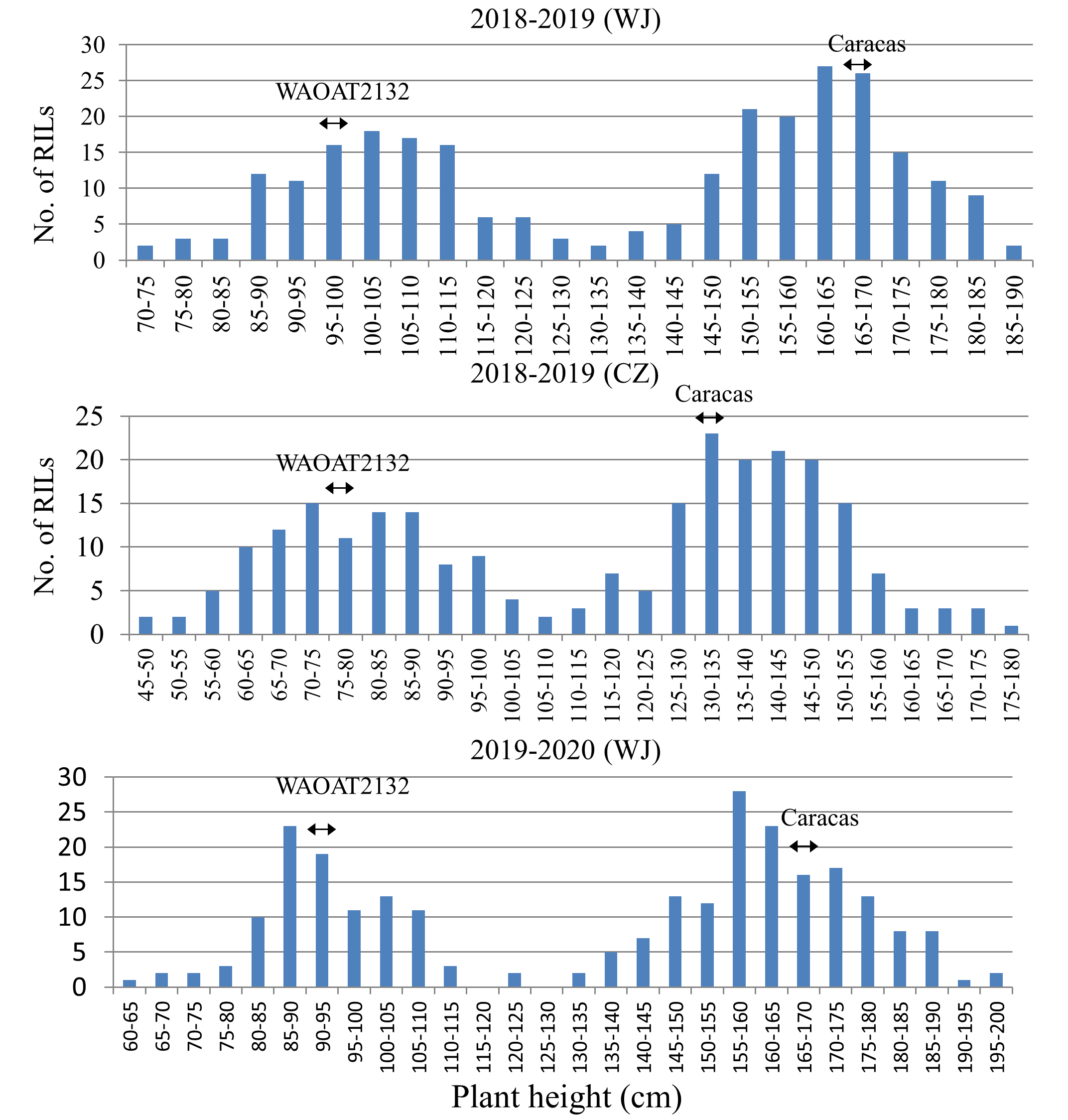


**Supplementary Figure 1.** Frequency distribution of plant heights in the WAOAT2132/Caracas RIL population across the three environments of testing.


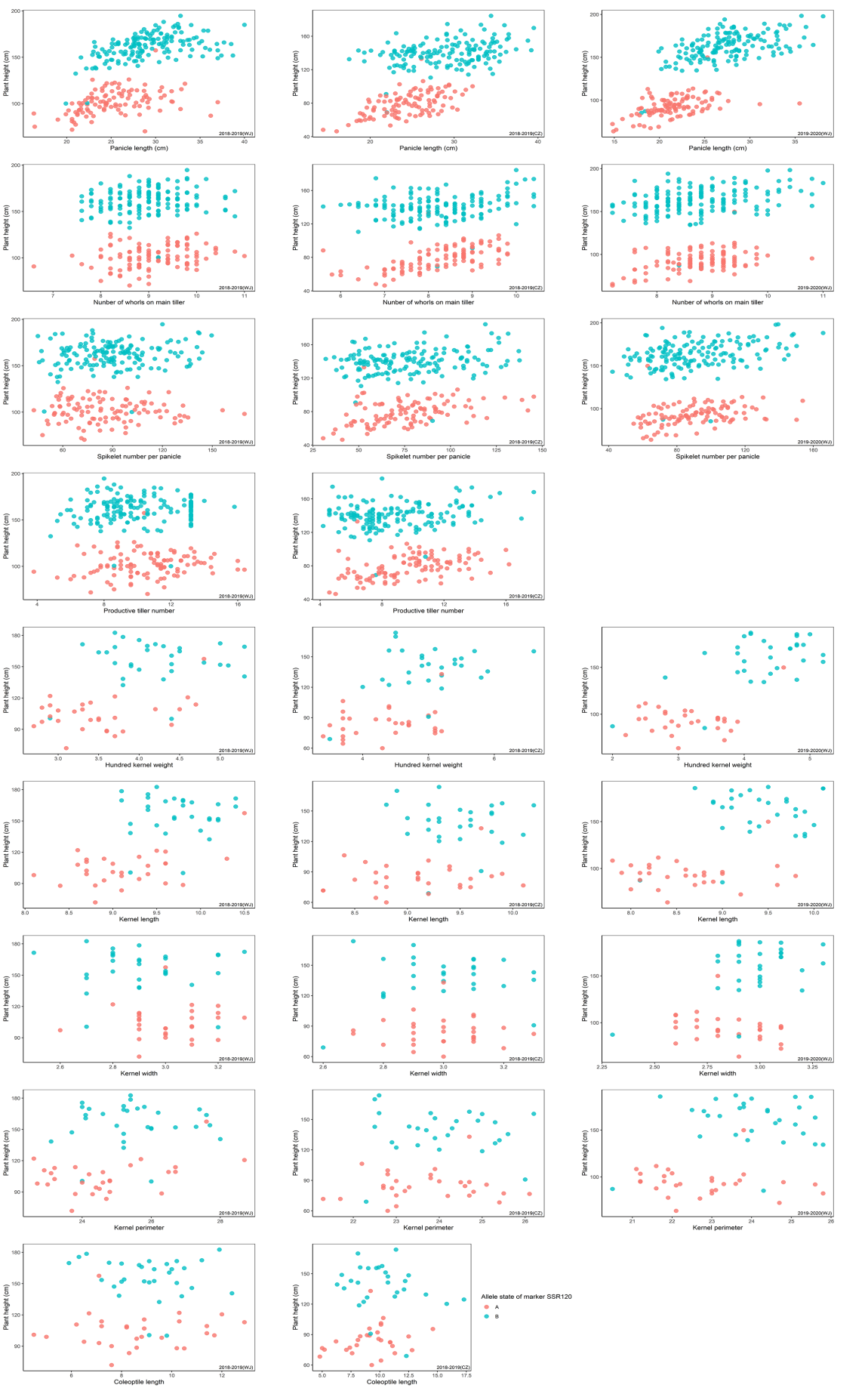


**Supplementary Figure 2.** Scatter plots displaying the relationships between plant height and other agronomic traits in the WAOAT2132/Caracas RIL population. Note, each line was colored according to the allele state of marker SSR120.


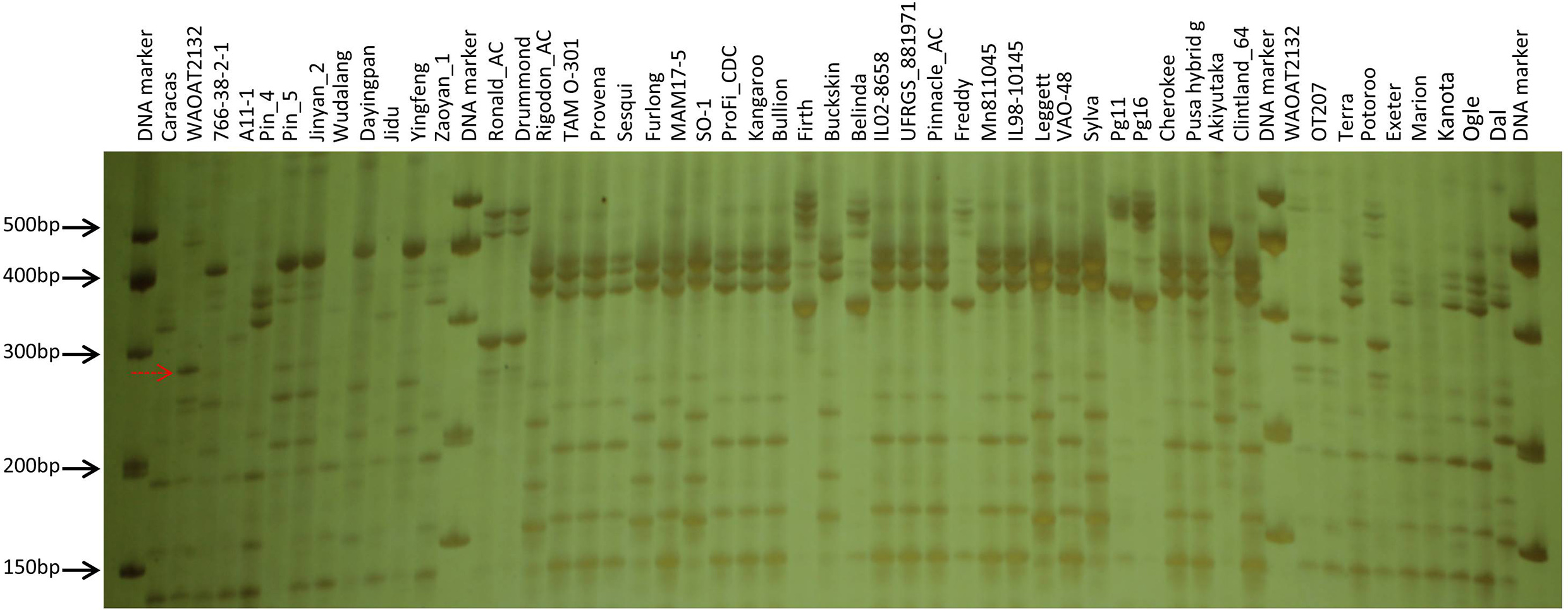


**Supplementary Figure 3.** Allele state of marker SSR120 in a set of diverse oat accessions. The red dotted arrow indicates the *Dw6* allele.

## Supplementary Tables

Table S1. List of the recombinant inbred lines and the parents used in this study and their allelic information of five polymorphic SSR markers and raw agronomic data in each of environment of testing.

Table S2. Allelic variation of marker SSR120 in 48 diverse oat accessions and 14 near-isogenic lines contrasting for *Dw6*.

Table S3. Primer information for microsatellite markers used in this study.
